# Optical Sensing with Simultaneous Electrochemical Control in Metal Nanowire Arrays

**DOI:** 10.3390/s101109808

**Published:** 2010-11-02

**Authors:** Robert MacKenzie, Corrado Fraschina, Takumi Sannomiya, Vaida Auzelyte, Janos Vörös

**Affiliations:** 1 Laboratory of Biosensors and Bioelectronics, ETH Zurich, Gloriastrasse 35, 8092 Zurich, Switzerland; 2 Laboratory for Micro-/Nano-technology, Paul Scherrer Institute (PSI), 5232 Villigen, Switzerland

**Keywords:** nanowire, sensing, nanoparticle, array, LSPR, electrochemistry, nanotechnology, biosensors, bioelectronics

## Abstract

This work explores the alternative use of noble metal nanowire systems in large-scale array configurations to exploit both the nanowires’ conductive nature and localized surface plasmon resonance (LSPR). The first known nanowire-based system has been constructed, with which optical signals are influenced by the simultaneous application of electrochemical potentials. Optical characterization of nanowire arrays was performed by measuring the bulk refractive index sensitivity and the limit of detection. The formation of an electrical double layer was controlled in NaCl solutions to study the effect of local refractive index changes on the spectral response. Resonance peak shifts of over 4 nm, a bulk refractive index sensitivity up to 115 nm/RIU and a limit of detection as low as 4.5 × 10^−4^ RIU were obtained for gold nanowire arrays. Simulations with the Multiple Multipole Program (MMP) confirm such bulk refractive index sensitivities. Initial experiments demonstrated successful optical biosensing using a novel form of particle-based nanowire arrays. In addition, the formation of an ionic layer (Stern-layer) upon applying an electrochemical potential was also monitored by the shift of the plasmon resonance.

## 1. Introduction

In this work we present various designs for nanowire arrays, their fabrication, their optical characterization and their potential in (bio-)electrochemical sensing applications. Existing combined electrochemical sensor systems, such as electrochemical optical waveguide lightmode spectroscopy (EC-OWLS) and electrochemical quartz crystal microbalance with dissipation (EC-QCM-D), have clearly demonstrated their individual uniqueness and usefulness [1–3]. Thus, the creation of a combined optical-electrical system designed for the nanoscale with noble metal nanowire arrays should offer a unique and powerful new platform, which will enable more complex chemical sensing and biosensing applications, as well as open new possibilities to explore the fundamental *in situ* behavior of nanowires. This will challenge and hopefully refute the misconception that only semiconducting nanowires are feasible for sensing.

Currently the emergent field of nanowire systems is strongly dominated by the work on semiconducting nanowires, with silicon nanowires in field-effect transistor (FET) configurations as the obvious current system of choice [1,4–6]. The favorable electrical properties of certain materials containing e.g., silicon [6–9], gallium [10], cadmium [11,12], titanium [13], or carbon (*i.e.*, carbon nanotube-based FET devices) [4,5] have yielded promising results in chemical sensing, biosensing and integrated electronics. Such nanowires represent very attractive bioelectrochemical transducer components, since their conductance is sensitive to surface perturbations induced by biochemical analytes [14,15]. Nanowires have earned so much attention that Roy *et al.* related nanowire-based systems to a paradigm shift in biosensing, but their focus seems only to extend to electrical sensing systems [4]. Proposed here is a new combination of sensing techniques at the nanoscale itself enabled by the design and implementation of arrays of gold and silver nanowires to harness the combined advantages of the wire array’s optical response and electrically conductive nature. In this work two types of large-scale noble-metal nanowire arrays, evaporated and particle-based, were fabricated.

Analogous to plasmonic nanoparticle or nanohole systems [16–18], plasmonic nanowires exhibit characteristic optical resonance around the visible wavelength, known as localized surface plasmon resonance (LSPR). The resonance wavelength and field strength is dependent on the material, size, shape, separation from other plasmonic structures (e.g., periodic gaps between nanowires) and surrounding medium. For biosensing, the adsorption of biomolecules within the enhanced field is detected as a resonance peak shift, since the adsorbed molecules have a higher refractive index than the surrounding solution. Compared to bulk metal, charge density oscillations in metallic nano-objects are confined in all directions where the structural dimension is significantly smaller than excitation wavelength [19]. Thus, nanoparticles demonstrate a case of total confinement, but nanowires are a special geometric case with a 1-D length axis that can be regarded as infinitely long compared to their width. Due to the missing confinement along the length axis, localized plasmon oscillations are restricted to the plane perpendicular to the length axis. Thus, LSP modes in nanowires can only be excited by the component of polarized light perpendicular to the wire [20].

Yet at present there is a fundamental lack of understanding of the exact and predictable response of nanowires or the response of environments surrounding nanowires upon stimulation. For example, little is known about the *in situ* changes in electron surface potential of the nanostructure material or the formation and effect of a Stern layer around a nanowire and how these phenomena exactly alter the optical or electrical response of the material. Although recent investigations of combined LSPR and electrochemistry with *in situ* cyclic voltammetry (CV) have been performed using gold nanoparticles immobilized on indium tin oxide (ITO) surfaces [18], as well as further studies combining metal nanoparticle-based LSPR with electrochemistry [21–25], all have electrically stimulated the entire active surface (*i.e.*, background and nanostructure), but none have isolated the electrical stimulation to only the nanostructure itself (*i.e.*, acting as a nanoelectrode on an insulated surface). This, however, is an inherent ability and advantage of a well-designed nanowire system.

The advantages of an array configuration, whether referring to a collection of single-nanowire devices or a collection of multiple-nanowire devices, include: (i) high uniformity and reproducibility, (ii) multi-analyte parallel sensing possibilities, (iii) high device yield with built-in redundancy to avoid failure, (iv) scalability and large-scale manufacturability, and (v) the direct integration into external systems (e.g., electrical, optical, mechanical).

Some nanowire arrays of various configurations for sensing applications or simulation already exist. The well-known work of Gao *et al.* describes the fabrication and use of a nanowire array to measure the surface-charge-induced resistance response upon DNA hybridization [15]. Their array is a small collection of individually addressable single silicon-nanowire FET devices and not, for example, an array of sub-arrays as defined in this work. Schider *et al.* have investigated the optical response and polarization dependence of silver and gold nanowire gratings, but without a sensing application [20]. Perhaps the closest known comparable nanowire system is from the work of Byun *et al.*, who attempt to simulate the LSPR sensitivity of a self-assembled monolayer (SAM) on noble metal nanowire arrays [26]. Their work was restricted to the simulation of nanowire arrays with large periods and the refractive index sensitivity of three different nanowire profiles made of gold or silver was investigated. By decreasing the period from 400 nm to 250 nm, a blue-shift of the LSPR wavelength was observed. Furthermore, their computer simulation calculated a predicted sensitivity of 16 nm/RIU for an adsorbed SAM layer on silver nanowire arrays and 17.4 nm/RIU for a SAM on gold. However, until now no experimental results have been found for LSPR-based (bio-)electrochemical sensing with metal nanowire arrays. Similar, yet alternative methods for self-assembled or particle-based nanowires can be found in the works of Blech *et al.* and Hung *et al.* [27,28].

## 2. Experimental Section

**Figure 1.**
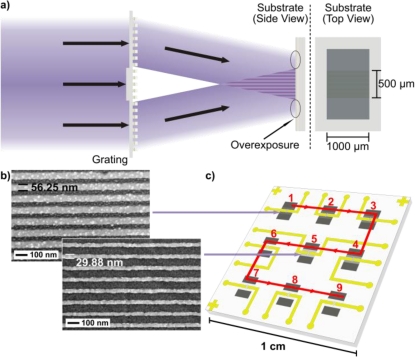
An illustration of a realistic EUV-IL exposure. A PMMA resist on an Nb_2_O_5_ coated wafer is exposed to light diffracted by an e-beam fabricated grating. The grating-dependent periodicity of the resulting interference pattern here is 100 nm. The result is a chip with nine exposed regions, which is illustrated in **(c)** as the end product of the complete nano- and microfabrication. The varying exposure times in each region yield nine regions with varying line widths. Region one has the widest openings in the PMMA (*i.e.*, the widest lines after fabrication) as shown in **(b)**, while region nine has the thinnest openings. The illustration **(c)** shows a typical nanowire fabrication in only five regions, since the dose of certain exposures is not strong enough to expose the photoresist to the substrate surface. Thus, the line pattern disappears upon photoresist removal, yielding no lines in such regions. On such a typical sample the thinnest continuous wires would exist in a middle region, such as region five.

The arrays in is this work were created using Extreme Ultraviolet Interference Lithography (EUV-IL) and exposed in PMMA resist [29,30]. Niobium oxide (Nb_2_O_5_)-coated glass wafers were used as the substrate for all fabricated nanowire arrays in this work. The negative charge of Nb_2_O_5_ is advantageous for the adsorption of positively charged polymers, such as PEI (polyethyleneimine), which is used later for fabrication self-assembled nanowire arrays. Compared to other oxides, Nb_2_O_5_ has exhibited the best pattern quality when applying the process of Molecular-Assembly Patterning by Lift-Off (MAPL) [31]. The glass wafers were exposed at the Paul Scherrer Institute (PSI, Laboratory for Micro- and Nano-technology). Although they have already demonstrated the fabrication of sub-10 nm line patterns with 50 nm periods [30], all nanowires used in this work have a fixed periodicity of 100 nm, unless otherwise stated. By altering the exposure time, the width of the nanolines (or correspondingly the separation in between) can be tailored. This nanoline fabrication process is summarized in Figure 1.

**Figure 2.**
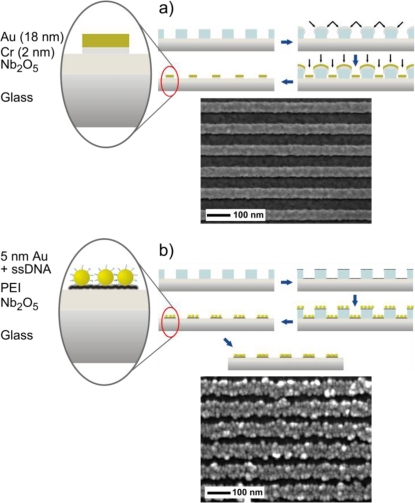
Continuous nanowires **(a)** are created through a process of shadow deposition where Cr is angularly evaporated as an intermediate layer and up to 50 nm of Au at normal incidence. Particle-based nanowires **(b)** are created in a process of Au nanoparticle adsorption to a PEI layer. In both processes the PMMA is removed with NMP (N-Methylpyrrolidone). The final step in image **(b)** illustrates the chemical enhancement of the gold nanoparticles, which is explained in more detail in the Appendix. The SEM Images show examples of the resulting nanowires from the corresponding fabrication method.

The resulting nanoline patterns are converted to two different systems of nanowires either with conventional material deposition (e.g., shadow evaporation) or with unconventional nano-object self-assembly (e.g., nanoparticles) as summarized in Figure 2 [14,32]. The evaporated nanowires more easily enable the combined monitoring of both the LSPR and the electrical signals due to their more standardized fabrication method. For particle-based nanowires it is a long-term goal to develop their bottom-up fabrication to ensure more reliable electrical conduction. However, due to their intriguing optical properties the LSPR response of the particle-based nanowire arrays is presented in this work, with no electrical stimulation.

The evaporation method results in continuous e.g., gold nanowires ranging from 20 nm (shortest successful exposure) to 90 nm (longest successful exposure) in width. Again, due to varying exposure doses, the nanowire width decreases from region 1 to region 9 (with decreasing exposure time), but not necessarily linearly. Furthermore, the exact nanowire widths slightly differ from production batch (e.g., wafer of chips) to production batch. The dimensions of the evaporated Au nanowires for this work are summarized in Table 1. Dependent on the height of the PMMA photoresist layer, the evaporated nanowires typically have total heights no more than 50 nm. The height of the evaporated gold and silver nanowires in this work was 15 nm with an additional adhesion layer of 2 nm chrome. This method requires cleanroom conditions, high vacuum and yields reliable, continuous wires.

The particle-based nanowires are created with DNA-coated gold particles (British Biocell), which adhere to a PEI layer on the Nb_2_O_5_-coated substrate within the PMMA template. Initially the particles are not dense enough for electrical conduction, thus they are still referred to as particle nanolines. A chemical process of gold enhancement (GoldEnhance, Nanoprobes, NY, USA) enlarges the particles with the intent to grow the particles into each other, thus completing the conducting wire structure. A successful time-controlled growth prevents the wires from growing into each other [14]. The widths of the particle-based nanowires are comparable to the evaporated nanowires, but the height of the nanowire depends mainly on the size of the nanoparticle and length of enhancement. Unless otherwise stated, the nanoparticles used in this work were 5 nm in diameter and the final heights of the gold nanolines and nanowires have been estimated with AFM at nearly 10 nm after 3–5 minutes of enhancement. Except for the creation of the EUV-IL nanolines this bottom-up, self-assembly approach does not require cleanroom conditions, vacuum, or extensive cleaning since the process takes place within a liquid interface. The electrical properties differ from the continuous nanowires, but the additional surface area could prove more advantageous and sensitive to surface interactions. Additionally, it would be possible to imbed other particles or nano-objects (e.g., carbon nanotubes) into the nanowire structure, thus tailoring the optical or electrical properties.

The arrays for this work differ in configuration to other referenced works, because each fabricated nanowire sensing region contains up to 5,000 multiple nanowires connected in parallel of up to 1 mm in length. Thus they more represent an array of large-scale nanowire arrays on a single chip [14,30]. This immediately enables the possibility of parallel, multi-analyte sensing in separate regions or even within a single nanowire region on one chip. Through microfabrication each nanowire sensing region (*i.e.*, a collection of parallel nanowires) is addressable, as seen in Figure 1(c). Here, the nano-/micro-fabricated optical chips were coated with S1805 photoresist, exposed and developed to create a window to the nanowires between the contact pads, in which the optical inspection was performed. This protects the contact pads and better ensures that only the nanowires are in contact with the surrounding medium. More information on the nano- and microfabrication of these arrays is provided in the Appendix.

All LSPR results here have been collected with transmitted light through the nanowire sample, thus permitting the observation of the extinction spectrum peak and intensity. The spectra were recorded by SpectraPro 2150 (PIXIS 400, Princeton Instruments, USA) using halogen lamp illumination. The recorded information was evaluated by a custom-made program. The peak position was determined by fitting the spectrum with a parabolic function.

## 3. Results and Discussion

**Figure 3.**
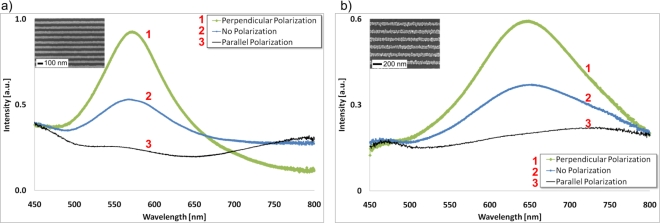
**(a)** A polarization dependent LSPR extinction spectrum of evaporated Au nanowires and **(b)** particle-based Au nanowires (200 nm period) in air. The most efficient excitation of localized surface plasmons can be achieved by perpendicular polarization with respect to the nanowire axis, but parallel polarized light results in almost no plasmon excitation.

Access to unconventionally powerful nanolithography to create nanoline templates enabled a dual-fabrication approach of two types of large-scale nanowire arrays with high periodicity and structure sizes in the tens of nanometers: the top-down evaporation of continuous metal (e.g., gold) nanowires or the bottom-up self-assembly of particle-based nanowires. Having achieved the necessary nano and microfabrication techniques to simultaneously expose the nanowire arrays to optical and electrical stimuli, this initial study focused on the characterization of the combined optical-electrical nanowire system, as well as its clear demonstration as a functioning sensing platform. This necessitated the combined knowledge from the fields of biology, chemistry, physics and engineering to explore the young and relatively unexplained concept of metal nanowire sensing. It is the intention that results shown here and future work with combined optical and electrical techniques will help to expand the understanding of electrical double layer formation, as well as the response of nanostructures to bio-electrochemical surface reactions. Although both types of nanowire arrays, evaporated or particle-based, exhibit a polarization-sensitive response, as demonstrated in Figure 3, all measurements in this work were performed with non-polarized light, unless otherwise stated.

**Figure 4.**
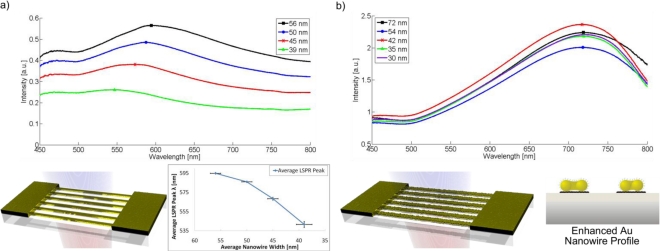
Optical extinction spectra of selected measureable chip regions with **(a)** evaporated Au and **(b)** 5 nm Au particle nanowires after chemical gold enhancement. The additional plot under the evaporated gold spectra shows the region-dependent LSPR peak of four different chips, thus demonstrating the spectral response dependence on nanowire width & separation for this system (*i.e.*, at a constant 100 nm period for all regions).

To further characterize the sensor performance of the nanowire arrays and to compare different sensor architectures, the bulk refractive index sensitivity *m_LSPR_* was determined by the relation Δ*λ* = *m_LSPR_* · Δ*n_medium_* in units of nm/RIU (Refractive Index Unit). It describes the resonance wavelength-shift Δ*λ* due to a change in the refractive index of the surrounding medium Δ*n_medium_* where no significant adsorption layer is formed. Glycerol is a common substance for the determination of *m_LSPR_* due to its non-adsorption and sufficient refractive index range at increasing weight percentages. Varying concentrations of 0% (ultrapure Millipore water), 20%, 40%, 60% and 80% by weight of glycerol were exposed to several geometries of nanowire arrays in order to investigate their bulk refractive index sensitivity. The extreme viscosity of 100% glycerol would prevent a successful injection into the flow cell and, thus, wasn’t attempted. The optical responses of evaporated gold and silver nanowire regions were measured, as well as a 5 nm and a 20 nm particle-based nanowire system before photoresist removal, after photoresist removal and after enhancement. Figure 4 provides a summary of the most important example geometries and their corresponding spectrum in air.

**Figure 5.**
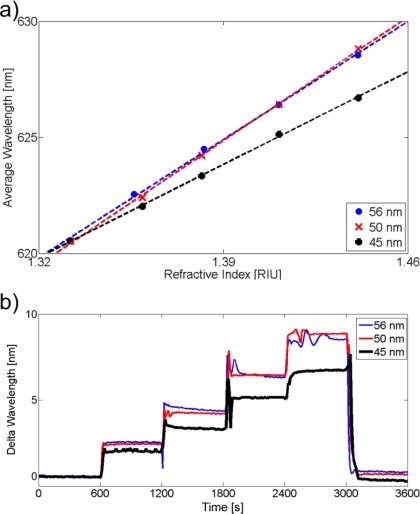
**(a)** The average peak resonance wavelength of three arrays of different nanowire widths on an evaporated Au sample as a function of the refractive index of the surrounding media. The slope corresponds to the bulk refractive index sensitivity, *m_LSPR_*. **(b)** The spectral position of the LSPR peak of an evaporated Au sample as a function of time for varying concentrations of glycerol with different refractive indices. The higher the refractive index of the surrounding medium, the higher the resonance wavelength. (The high pressures in the flow cell and/or air bubbles during medium exchanges could account for the strong fluctuations of the signal just after each new injection of glycerol, especially pronounced at a glycerol level of 80%).

Upon establishing a stable baseline in ultrapure water, 1.5 mL of solution (∼7.5 times the volume of the flow cell) was manually pumped into the cell at each injection step. Finally, the flow cell was rinsed by at least 5 mL of ultrapure water. Consistent with theory, the plasmon resonance wavelength increases as the refractive index increases. Rinsing with ultrapure water nearly re-initializes the system to the baseline, thus indicating a successful medium exchange. The average wavelength in each interval was then calculated and the refractive indices of the mentioned glycerol concentrations were measured by a J357 automatic refractometer (Rudolph Research Analytical, USA). As predicted by Δ*λ* = *m_LSPR_* · Δ*n_medium_* an almost linear dependence can be observed when the average peak wavelength is then plotted as a function of the refractive index of the surrounding medium. The resulting bulk refractive index sensitivity and an example glycerol measurement with evaporated gold nanowires are displayed in Figure 5. An overview of all relevant system sensitivities is provided in Table 2.

By optimizing the tracking algorithm of the monitored LSPR spectrum, it is possible to maximize the sensitivity or to minimize the limit of detection (LOD), which is calculated by 3*σ*/*m_LSPR_*, where σ donates the standard deviation of the baseline signal over 500 seconds. The highest obtained sensitivity value was 114.6 nm/RIU for an evaporated gold nanowire region (width: 56 ± 2 nm; height: 2 nm Cr + 15 nm Au), but this didn’t ultimately result in the best LOD, which was shown to be detectable to the fourth digit of the refractive index. A comparison to a similar nanowire array system could not be made, since no known experimental results exist in literature. Chen *et al.* reports bulk sensitivities from 44 nm/RIU (nanospheres) to 703 nm/RIU (nanobranches) in particle systems with similar glycerol measurements [33]. Bulk sensitivities as high as 880 nm/RIU were reported for nanoring structures by Larsson *et al*. [34]. Thus the measured sensitivities for the nanowire arrays are modestly comparable, while still possessing the advantage of simultaneous electrical conduction. Regarding the material dependence for nanospheres it is understood that the LSPR response of silver nanoparticles is more intense and extends to a greater distance than gold, however, partially due to the complex geometry of the tested nanowire array and the oxidation of silver, the gold nanowires display a much higher sensitivity and a better LOD [35,36]. Furthermore, for wire systems of these dimensions, similarities can be seen in comparison to the LSPR refractive index sensitivity of gold nanorod biosensing systems [37].

**Figure 6.**
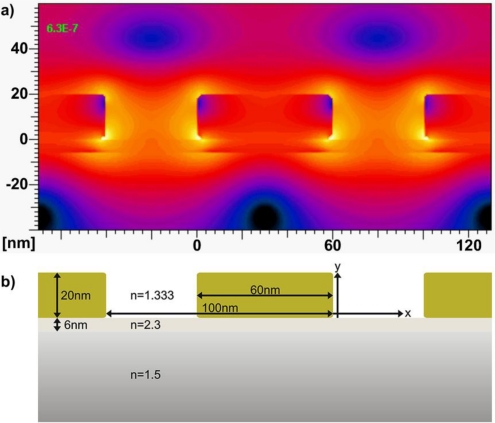
**(a)** A sample of the simulated time-averaged electric field distribution for the cross-section of a nanowire array at the resonance wavelength of 633 nm in water. Such simulations approximate well the field strength, the field decay length and resonance response to compare and to confirm bulk sensitivities with the measured results. The electric field is especially concentrated at the corners of the wires and extends notably into Nb_2_O_5_ the substrate. All nanowire cross-sectional dimensions and refractive indices are noted in **(b)**. The Cr layer between Nb_2_O_5_ and Au has been neglected.

Simulations offer a deeper understanding into the further characterization of the nanowires arrays, as well as their sensing limits and potential for optimization. A simulation of the field decay length and the extinction spectrum was performed by the multiple multipole program (MMP) using the MaX-1 software package [38]. Various geometries (e.g., width, height, rounding of the corners) have been simulated and compared to experimental data. Figure 6 graphically illustrates the simulated time-averaged electric field distribution at resonance (633 nm in water) of a structure that matched best with the experimentally measured evaporated nanowire systems listed in Table 2. For a wire system with similar dimensions (width: 60 nm; height: 20 nm) to existing nanowires (see Table 1), the simulation predicts a bulk refractive index sensitivity of 104 nm/RIU compared to the maximum measured value of 114.6 nm/RIU. A strong field enhancement can be observed between the nanowires. As expected, the field enhancement is especially pronounced at the corners of the rectangular wires. The field decay lengths at the positions of maximum field enhancement was approximated between 4.2 nm and 6.0 nm. Both extracted values are reasonable for an LSPR-based sensor system [39,40].

**Figure 7.**
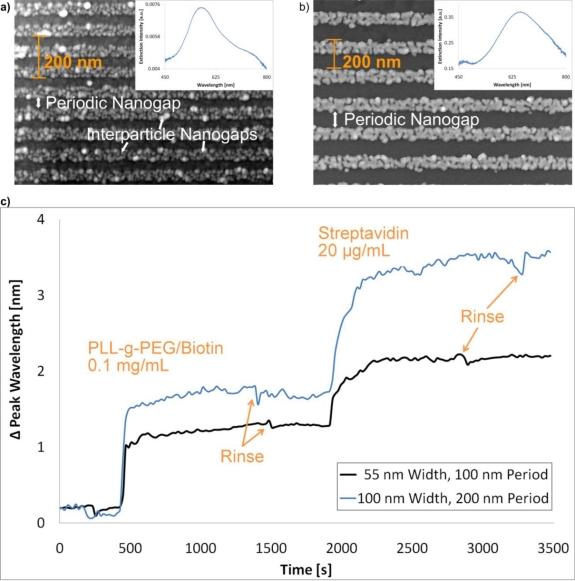
SEM images of a chemically enhanced particle-based nanowire arrays with a line period of **(a)** 100 nm or **(b)** 200 nm, produced with 5 nm enhanced Au particles. Inserted is the corresponding extinction spectrum of each array, but the spectrum for the 200 nm period array was obtained with perpendicularly polarized light. By tracking the LSPR response with a spectrometer in transmission mode, the peak shift of the extinction spectrum was monitored. A shift in wavelength on the order of ∼1.2 nm yielded 3σ-calculated signal-to-noise ratios near 30.

Initial biosensing experiments were performed on Au particle-based nanowire arrays using a standard streptavidin-biotin affinity model to demonstrate the feasibility. By capitalizing on the LSPR in the particle-based nanowire array, it was possible to observe changes in the extinction spectrum as the first layer of biomolecules adsorbed to the surface of the exposed gold and surrounding Nb_2_O_5_ surface, as shown in Figure 7. The baseline was obtained in a HEPES buffer (10 mM 4-(2-hydroxyethyl)-1-piperazineethanesulfonic acid, 150 mM NaCl, pH = 7.4). Then 0.1 mg/mL of PLL-g-PEG/Biotin (biotin-derivatized Poly(l-lysine)-*graft*-poly(ethylene glycol)) was injected into the flow cell, allowed to adsorb and coat the surface [41]. Upon buffer rinsing, 20 μg/mL of streptavidin was injected, allowed to bind to biotin and then subsequently rinsed. With peak shifts of ∼1.2 nm and a 3σ noise of 0.04 nm, a first-attempt signal-to-noise ratio of 30 was obtained for the array of 100 nm period. The 200 nm nanowire array was measured with perpendicularly polarized light. Despite larger peak shifts, there was also a larger noise than in the 100 nm nanowire array signal. The streptavidin (SA) and PLL-g-PEG/Biotin solutions were also prepared in the HEPES buffer. Here the change in the extinction spectrum is determined by a shift in peak position.

The combination of electrical and electrochemical properties of the nanowire arrays with their optical response is unique. As mentioned earlier, there are no known studies on the *in situ* combination of LSPR and electrochemistry with nanowires. Furthermore, the nanowire array is the first such system where the applied currents and potentials are confined only to the working electrode (*i.e.*, the nanowire on an insulating background) instead of to the entire active surface (*i.e.*, conductive background and nanofilm or nanoparticle). This isolates the electrochemistry to the working electrode and eliminates background effects. Thus, these nanowire arrays are the best-known candidate to link any possible electrochemical-based optical and electrical interdependencies for nanoscale objects, such as the effect of local refractive index changes and/or the formation of an electric double layer on the spectral response.

**Figure 8.**
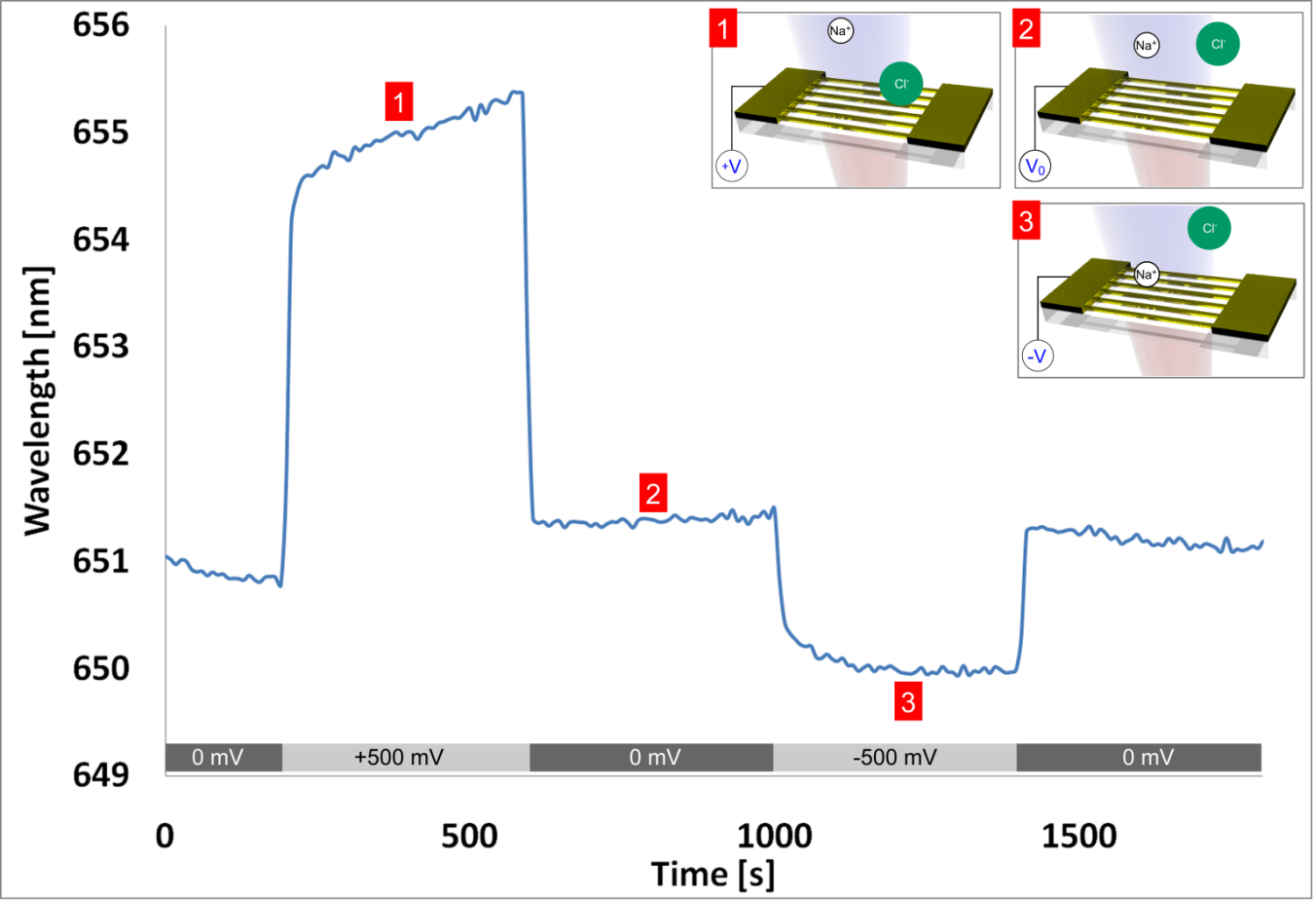
In a 150 mM NaCl solution, voltages of ±500 mV were applied to the nanowire array (*i.e.*, working electrode) and regulated by a silver reference electrode and platinum counter electrode. The individual nanowires had widths of 58 nm and heights of 2 nm Cr + 15 nm Au. As illustrated in the inset images, voltages attract the charged ions of opposite polarity to the surface, thereby changing the local refractive index. This change in refractive index is seen as a corresponding shift in the LSPR wavelength of the nanowire array.

To investigate such a local electrochemically induced refractive index effect, the substrate was mounted in an electrochemical-optical flow cell (see Appendix) with a platinum counter electrode and a chlorinated silver wire as semi-stable reference electrode. Potentiometric measurements were performed with a potentiostat (Model 2053, Amel Instruments, Italy). This configuration enables the simultaneous collection of the LSPR-based nanowire spectral response data, the control and monitoring of the electrochemical cell, as well as the measurement of the nanowire electrical properties across the array (e.g., nanowire array resistance). According to the DLVO theory, the applied potential on the surface forms an electric double layer in solution [42–44]. The double layer consists of the Stern layer at the immediate metal-solution interface and the diffusive (Gouy-Chapman) layer in the solution phase. The application of voltages to the nanowire array attracts and repels ions in solution, thereby changing the local refractive index near the wires, which is observable in the LSPR spectral response. Visible in the measurement shown in Figure 8 is an outstanding wavelength shift of over 4 nm at +500 mV, as well as polarity dependence in 150 mM NaCl solution.

**Figure 9.**
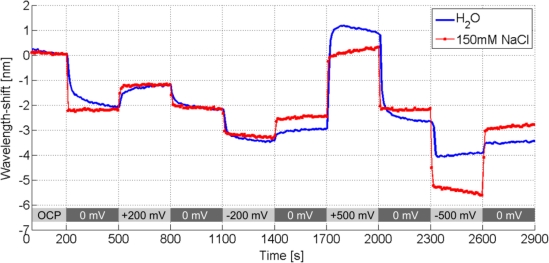
The voltage-dependent optical response of Au nanowires (width: 50 nm, height: 2 nm Cr + 15 nm Au) in an electrochemical flow cell in solutions of differing ionic strength. At the beginning of the measurement (<1,400 s) the optical response in 150 mM NaCl is almost step-like compare to the response in ultrapure water, where the response is slower because of the low concentrations of (naturally existing) ions e.g., OH^−^, ${\text{CO}}_{3}^{-}$, or H+. At voltages of more than −200 mV in water the unprotected silver reference electrode began to coat the nanowire array with silver ions, thus altering the measurement baseline and optical response.

Since a chloride ion has a higher polarizability (3.65 Å^3^) than a water molecule (1.45 Å^3^), a red shift of the resonance is anticipated when the ions are attracted to the gold surface at positive potentials [45]. The inverse holds true for sodium ions, which have a lower polarizability (0.086 Å^3^) [45]. The dependence of the response time to ionic strength is shown more clearly in Figure 9 and has been extensively studied by Sannomiya *et al.* with gold nanoparticle surface reactions in NaCl [17]. Figure 9 also shows a voltage dependence of the refractive index, observable as a corresponding LSPR wavelength shift.

## 4. Conclusions

The measurable LSPR-based sensing performance of noble metal nanowire arrays beyond initial theoretical predictions has been shown. As a first step towards biosensing, the use of nanofabricated particle-based nanowire/nanoline arrays to measure the adhesion of biomolecules has been demonstrated. Furthermore, the unique system configuration and LSPR-based sensing principle enabled simultaneous *in situ* optical and electrical measurements and electrochemical control for the study of local refractive index changes. LSPR peak shifts of over 4 nm, a system sensitivity up to 114.6 nm/RIU (Refractive Index Unit) and limits of detection as low as 4.5 × 10^−4^ RIU have been obtained for evaporated nanowire arrays.

In this work it is left undetermined if the LSPR peak shift in ionic solutions occurs only in response to a local change in refractive index in the medium surrounding the nanowires. However, the constructed system with combined optical and electrical techniques should offer new possibilities to study electrical double layer formation and surface reactions near and/or on the nanowires. This future study using nanowires is already supported by the work of Sannomiya *et al.* for nanoparticle systems and the formation of a surface chloride film on noble metals at anodic potentials, which has been observed by ellipsometry and quartz crystal microbalance (QCM) [17,46].

Future measurements will focus on the use of polarized light to combine optical measurements with cyclic voltammetry (CV) to provide complementary information about the surface reactions and their effect on the properties of the nanowires. Furthermore, this system still possesses an enormous potential for improvement. Alone the controlled modification of the wire materials, wire profiles, wire gaps, amount of material, as well as the functionalization of the wires stirs the imagination regarding the yet unknown and achievable limits of detection and application.

## Figures and Tables

**Table 1.** A summary of mean nanowire (NW) widths for the evaporated Au & Ag nanowire arrays used in this work. The values are based on the estimated mean values of SEM images of four Au chips and two Ag chips from the same production batch. In height, all nanowires have a 2 nm adhesion layer of chrome and 15 nm of gold or silver.

**Region**	**Evaporated Au NW Width [nm]**	**Evaporated Ag NW Width [nm]**
1	56 [±2]	50 [±2]
2	50 [±2]	44 [±2]
3	45 [±2]	41 [±2]
4	39 [±3]	36 [±3]
5	32 [±3]	28 [±3]
6	27 [±3]	No line structure
7	No line structure	No line structure
8	No line structure	No line structure
9	No line structure	No line structure

**Table 2.** A summary of bulk refractive index sensitivities and limits of detection (LOD) for various nanowire/nanoline geometries and materials. The headings Region 1 & Region 2 refer to the measured nanowire region on a chip. Please refer again to Table 1 for the summarized wire dimensions. An ‘x’ is displayed in regions where measurements were either not yet performed or not reliable.

**Material System**		**Bulk Sensitivity [nm/RIU]**		^*^LOD [RIU]	
		Region 1	Region 2	
Evaporated Au		114.6	82.8^*^	4.5·10^−4^
Evaporated Ag		47.9^*^	x	4.1·10^−3^
5 nm Au NP	Before Removal	34.8^*^	28.8	2.4·10^−3^
	After Removal	33.3	36.1^*^	2.0·10^−3^
	After Enhancement	15.6	17.3^*^	1.5·10^−2^
20 nm Au NP	Before Removal	53.0^*^	48.0	1.0·10^−3^
	After Removal	49.3	57.4^*^	8.1·10^−4^
	After Enhancement	x	18.8^*^	5.6·10^−3^
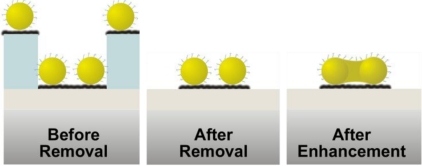				

**Figure A1.**
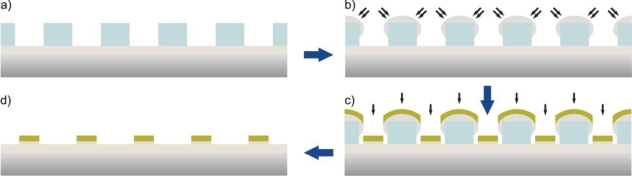
**(a)** PMMA line pattern fabricated by EUV-IL, **(b)** Cr evaporated at an incident angle of 15°, **(c)** Evaporation of Cr as intermediate layer and up to 50 nm of Au at normal incidence, **(d)** Lift-off of the PMMA lines by NMP (N-Methylpyrrolidone) or acetone.

**Figure A2.**
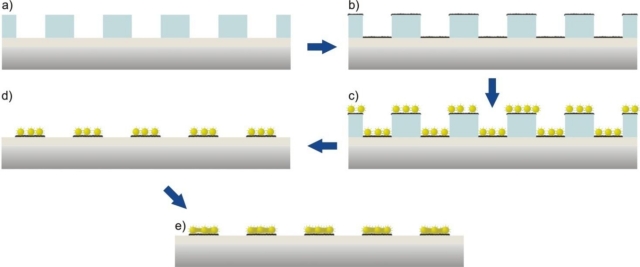
**(a)** PMMA line pattern fabricated by EUV-IL, **(b)** Adsorption of PEI, **(c)** Adsorption of gold nanoparticles to the PEI layer, **(d)** Chemical enhancement of the gold nanoparticles, **(e)** Lift-off of the PMMA lines by acetone.

**Figure A3.**
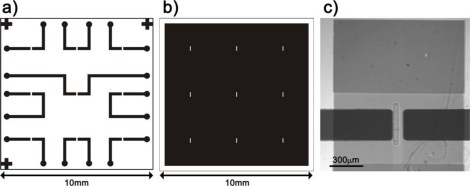
**(a)** Contact lead mask. A negative photoresist was used and those regions unexposed to light were afterward removed in the developer solution. **(b)** Window mask. For each nanowire array, one small window was opened by this mask. Note that this time a positive photoresist (e.g., S1805) was used. **(c)** Microscope image of a nanowire array. The window to the nanowires is placed between the two contact leads. The leads are separated by 100 μm, have a width of 200 μm and thus are potentially connected to 2,000 individual nanowires (100 nm period).

**Figure A4.**
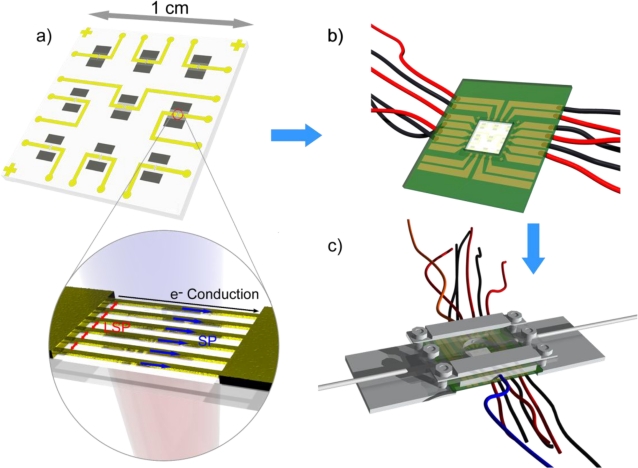
An illustration of the concept of an array of nanowire sub-arrays and microfabrication configuration. Image **(a)** shows multiple nanowire regions on a glass chip, where each connected region is itself an array of gold nanowires connected in parallel between gold contacts, as shown in image **(a)**. The integration of the optical chip into a PCB is shown in **(b)**, while **(c)** illustrates the entire chip’s integration into a transparent electrochemical flow cell. Also illustrate are the several sensing phenomena of this combined system: Sensing with surface plasmons (possible propagation along the wire axis), localized plasmons (confined to the perpendicular plane and the coupling between nanowire gaps), and electrical signals through the nanowires between the contact pads.

**Figure A5.**
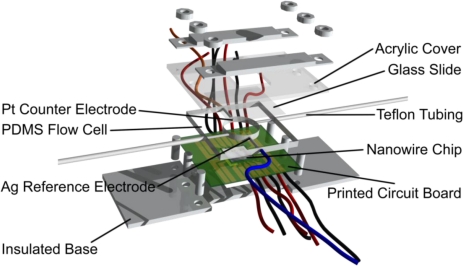
A custom built electrochemical flow cell in its disassembled state. The flow cell itself consists of PDMS which is pressed against the PCB and sealed on the upper side by a glass slide. The flow chamber has a cylindrical shape (height: 2 mm, diameter: 11 mm) with a total volume of roughly 200 μl. Two 0.8 mm Teflon tubes were inserted into two adjacent flow channels of the cell while a silver reference and a platinum counter reference electrode was placed into the remaining channels for electrochemical measurements.

**Figure A6.**
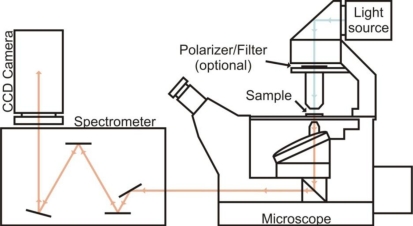
optical measurement setup in transmission mode. Light is guided through a nanowire array into a spectrometer. Finally, the transmitted spectral intensity is recorded by a CCD camera.

## Appendix

### Nanofabrication

Extreme Ultraviolet Interference Lithography (EUV-IL) is a newly emerging nanolithography method that combines the advantages of a parallel fabrication process with high resolution. These features make it an attractive tool for researchers who are increasingly in need of nano-patterning capability that is beyond what is available from other methods such as photolithography, e-beam lithography (EBL), and scanning probe lithography, in terms of resolution or throughput. The used EUV-IL setup is part of the X-ray Interference Lithography (XIL) beamline of the Swiss Light Source (SLS) [29,47].

The spatially coherent incident beam from an undulator is diffracted by a diffraction grating and the resulting fringe pattern produces lithographical patterning of resist on a wafer. The synchrotron light has a wavelength of 13.4 nm. The diffraction grating was fabricated by e-beam lithography and subsequent dry etching of a thin chromium film on top of a 100 nm thick silicon nitride support membrane, although EUV-IL itself can be used to create subsequent masks. Detailed information about the fabrication of the diffraction grating is provided by Auzelyte *et al.* [30]. The frequency of the resulting fringe pattern on the wafer is twice the frequency of the grating. Prior to exposure, we have thermally evaporated 6 nm niobium oxide (Nb_2_O_5_) on glass wafers and spin coated a 40 nm thick PMMA film on top. This PMMA film was developed after the EUV-IL process in MIBK:IPA (1:3) solution.

Two different approaches for the fabrication of nanowire arrays were used: metal evaporation and a particle-based self-assembling process. The first process is schematically shown in Figure A1.

At the beginning, chromium was evaporated while the glass wafers were tilted by 15°. This forms a structure on top of the PMMA lines that prevents the subsequent deposited metal at normal incidence from covering the whole exposed substrate. In the following step 2 nm chromium and 15 nm gold were evaporated at a rate of 1 nm/s at a pressure of 2 × 10^−6^ mbar. The resulting line width of the gold wires is smaller than the gaps between the PMMA lines due to a shadowing effect. This under-cut profile is necessary for the final PMMA lift-off. The nanowire quality mainly depends on the resist pattern roughness, the properties of the metal and the conditions for metal deposition.

The second approach is a self-assembling process of nanoparticles. All process steps are schematically depicted in Figure A2.

After cleaving the glass wafers into chips (9 potential regions of nanowire arrays per chip), each chip was rinsed in ultrapure water (18.2 MΩ·cm @25 °C, Milli-Q, Millipore Corporation, Switzerland) and treated for typically 10 seconds in oxygen plasma. Afterward the chip was covered by PEI (polyethylenimine) for 20 minutes. The adsorption of PEI onto Nb_2_O_5_ forms a positively charged layer which is the base for the adsorption of negatively charged metallic nanoparticles. We have used gold colloid solution purchased from BBInternational, UK with particle diameters of 5, 10 and 20 nm. The interparticle distance of the colloid/water solution was adjusted by tagging the nanoparticles with ssDNA thiol-5′-CCC-CCT-TAC-GAT-TCC-AT-3′ (Eurogentec, US), adjusting the salt concentration and centrifugation similar to a process described by Storhoff *et al*. [48]. The chips were then immersed for 18 hours in the concentrated DNA-gold colloid solution and afterward carefully cleaned by ultrapure water and dried with N_2_. The PMMA photoresist is then removed by immersion in 100% NMP for 1 hour including 10 minutes of ultrasonic cleaning. Despite a dense adsorption from the concentrated DNA-gold colloid solution, the density of adsorbed nanoparticles was still too low to support an electrical current through the particle nanowires. In order to connect the individual nanoparticles to a wire like structure, they were chemically enhanced. A droplet of 50 μl GoldEnhance^tm^ EM solution (Nanoprobes, US) was placed on the nanowire arrays for a maximum of 10 minutes. This was followed by a cleaning step in ultrapure water to stop the chemical enhancement.

### Microfabrication

In order to establish an electrical connection to the nanowire arrays, contact leads (e.g., width of 200 μm) were fabricated by conventional photolithography. The substrate was covered by a negative photoresist (ma-N 1400), spin coated at 3,000 RPM for 30 seconds and hardened by a soft-bake step for 2 minutes at 100 °C. Mask alignment and exposure were performed in a Karl Süss X380 mask aligner for 120 seconds followed by a 1 hour long relaxing of the resist at room temperature.

After removing the unexposed photoresist by immersion in ma-D 533/S developer for 120 seconds, 10 nm titanium and 40–50 nm gold were thermally evaporated. The photoresist was removed in 100% NMP. In a second photolithography step, the whole chip was again covered with S1805 photoresist and only one 50 μm × 300 μm small window per nanowire array was opened. This step isolates the contact leads and most of the nanowire array, but opens a window to only a portion of the nanowire array between the contact pads for measurement. A summary is shown in Figure A3.

Finally, specialized PCBs were fabricated to establish an electrical connection to the nanowire arrays, while still allowing optical interaction with the nanowire arrays. The contact leads were connected to the conductive paths of an inverted PCB by silver paste. Thus, the top-side of the chip and the windows to the nanowire arrays were completely separated from the electrical side of the PCB. Afterward the system was sealed by adding epoxy to the PCB/chip interface. The smooth back-side of the PCB was optimal for sealing with a PDMS flow cell. The complete configuration is summarized in Figure A4.

### Flow cell

In order to expose the nanowire arrays to various fluids, a reusable flow cell was fabricated. A schematic of the assembled and disassembled flow cell is shown in Figure A5.

### Measurement System

Optical measurements were performed in an Axiovert 200 Microscope from Carl Zeiss, Germany. Light from a halogen lamp is guided through a condenser lens further through the nanowire array under investigation and focused by a 5x objective into a SpectraPro 2150 spectrometer (Princeton Instruments, US). The spectra were recorded by a PIXIS500 CCD camera (Princeton Instruments, USA). All optical experiments were performed in this transmission geometry, as illustrated in Figure A6.

Prior to each measurement, a background and a flat field image were recorded in order to remove artifacts from distortions in the optical path and variations in the sensitivity of the CCD camera. During the measurements, all fluids were manually pumped through the flow cell by syringes. The spectral intensity (five integrations) along a line within the opened photoresist window was recorded by WinSpec/32 software. Finally, the sequence of spectra was evaluated by custom-made software, which determined the position of the LSPR peak in each spectrum with different fitting functions.
